# Personality, Stress, and Intuition: Emotion Regulation Abilities Moderate the Effect of Stress-Dependent Cortisol Increase on Coherence Judgments

**DOI:** 10.3389/fpsyg.2020.00339

**Published:** 2020-02-27

**Authors:** Elise L. Radtke, Rainer Düsing, Julius Kuhl, Mattie Tops, Markus Quirin

**Affiliations:** ^1^Department of Psychology, Osnabrück University, Osnabrück, Germany; ^2^Developmental and Educational Psychology Unit, Leiden University, Leiden, Netherlands; ^3^Department of Psychology, Technical University of Munich, Munich, Germany; ^4^PFH Private University of Applied Sciences, Göttingen, Germany

**Keywords:** emotion regulation abilities, stress regulation, cortisol, trier social stress test, coherence judgments

## Abstract

**Objective:**

Findings on the relationship between hypothalamus-pituitary-adrenocortical (HPA) activity and cognitive performance are inconsistent. We investigated whether personality in terms of emotion regulation abilities (ERA) moderates the relationship between stress-contingent HPA activity and accuracy of intuitive coherence judgments.

**Method:**

ERA and cortisol responses to social-evaluative stress as induced by a variant of the Trier Social Stress Test were measured in *N* = 49 participants (32 female, aged 18 to 33 years, *M* = 22.48, *SD* = 3.33). Subsequently, in a Remote Associates Task they provided intuitive judgments on whether word triples, primed by either stress-reminding or neutral words, are coherent or not.

**Results:**

Under relative cortisol increase participants low in ERA showed reduced performance whereas individuals high in ERA showed increased performance. By contrast, under conditions of low cortisol change, individuals low in ERA outperformed those high in ERA.

**Conclusion:**

Personality can moderate the link between stress and cognition such as accurate intuition. This can happen to a degree that existing effects may not be become apparent in the main effect (i.e. without considering personality), which highlights the necessity to consider personality in stress research, ERA in particular. We discuss the findings with respect to individual differences in neurobehavioral mechanisms potentially underlying ERA and corresponding interactions with cognitive processing.

## Introduction

Stress can strongly influence individuals’ emotions (Lazarus and Folkman, 1984) and cognitive proficiency (McEwen and Sapolsky, 1995; Lupien et al., 2005). Still, unless stress levels are excessively high, individual differences may become apparent or even crucial: Whereas some individuals show adequate performance in the absence of stress, they may choke in the presence of stress. By contrast, other individuals flourish under the presence of stress but show reduced performance under the absence of stress (e.g. Henderson et al., 2012; Dolcos et al., 2014). A pivotal variable that may moderate this effect are individual differences in emotion regulation abilities (ERA; Kuhl, 2000; Quirin et al., 2011b; Koole et al., 2012; Düsing et al., 2016).

Whereas individual differences in strategies of emotion regulation may be described in terms of reappraisal or suppression (Gross and John, 2003), ERA refer to individual differences in the general capability to autonomously and efficiently regulate negative affect and disengage from concomitant thoughts (e.g. Quirin et al., 2011b). Accordingly, individuals with high levels of ERA show high efficiency and success in emotion regulation defined as the self-regulated change of an affective response to a salient stimulus in order to adapt to external or internal demands, standards, or goals (Kuhl, 2000; Cole et al., 2004; Ochsner and Gross, 2005; Koole, 2009; Gross et al., 2011).

Emotion regulation abilities can be conceived of an important personality variable that lies at the core of action versus state orientation (Kuhl and Beckmann, 1994). Specifically, action-oriented individuals (high ERA) are better able to cope with demanding situations or failure and concomitant negative affect and thus stay capable of pursuing their goals. By contrast, state-oriented individuals (low ERA) show a tendency to remain in the state of their current, for example, negative, affect and thus tend to ruminate and worry (Kuhl and Beckmann, 1994; Kuhl, 2000).

In general, ERA as assessed by individual differences in action orientation predicted a number of cognitive and behavioral outcomes (Koole et al., 2012). Specifically, under high levels of demand, high ERA compared to low ERA individuals showed increased life balance (Gröpel and Kuhl, 2009), were more successful in semiprofessional sports (Heckhausen and Strang, 1988), in a classical Stroop paradigm (Jostmann and Koole, 2007; Ruigendijk and Koole, 2014), and in a working memory task (Jostmann and Koole, 2006). Furthermore, high ERA individuals excelled in conflict-loaded trials of the Tower of Hanoi task where goal-subgoal conflicts had to be solved (Jostmann and Gieselmann, 2014) and suffered less from ego-depletion effects on attention (Gröpel et al., 2014). By contrast, in all these tasks low ERA individuals showed performance drops or absent changes.

Whereas high ERA individuals tend to keep or even show elevated levels of performance under conditions of relatively high demand, low ERA individuals tend to perform better under conditions of relatively low demand (Koole et al., 2012), especially when receiving external support in these situations, for example by visualizing an accepting person (e.g. Koole and Jostmann, 2004; for a review see Koole et al., 2005). Similarly, stress-induced drop in well-being observed in low ERA individuals was buffered when they were personally valued, or a sense of relatedness with others was primed (Chatterjee et al., 2013). Low ERA individuals also showed reduced cortisol responses to social stress after the application of intranasal oxytocin, suggesting that oxytocin might buffer endocrine stress responses in low ERA individuals (Quirin et al., 2011b). To summarize, high ERA individuals are able to down-regulate negative affect and thereby maintain or improve their performance in cognitive tasks under high demands or stress. By contrast, low ERA individuals can outperform high ERA individuals under low stress conditions or when receiving external support.

A large literature demonstrates how cognitive performance can be influenced by stress and that it correlates with endocrine stress markers such as hypothalamus-pituitary adrenocortical system activity and concomitant cortisol release. In primate brains, glucocorticoid receptors being a determinant for the cortisol performance relationship (Lupien et al., 2005) have been shown to be present in the hippocampus but to be more prominent in the prefrontal cortex and thus not only hippocampus but also the frontal lobes are affected by cortisol increase (Patel et al., 2000; Vogel et al., 2016). In line with this, acute stress as induced by a group version of the TSST was associated with increased decision-making competence (Shields et al., 2016) and a meta-analysis (Shields et al., 2015) showed that short-term effects of stress as indicated by stress-contingent cortisol increase included enhanced response inhibition and working memory impairment while long-term effects were reversed.

Glucocorticoid increase and performance relationship appears to follow an inverted u-shaped function. This is due to the tenfold affinity of mineralocorticoid compared to glucocorticoid receptors affinity to bind cortisol (Reul and de Kloet, 1985) which leads to different molecular cascades (cf. de Kloet et al., 1999; de Kloet et al., 1998), which in turn lead to the different performances. That is, very low and very high levels of circulating cortisol lead to a dampened performance, while medium levels lead to an improved performance in memory tasks (Lupien et al., 2005). Furthermore, Lupien et al. (2005) concluded that the specific shape and shift of the function, that is, the points of performance increase, optimal level and performance decrease varies between individuals as a function of the number of glucocorticoid and mineralocorticoid receptors.

**FIGURE 1:**
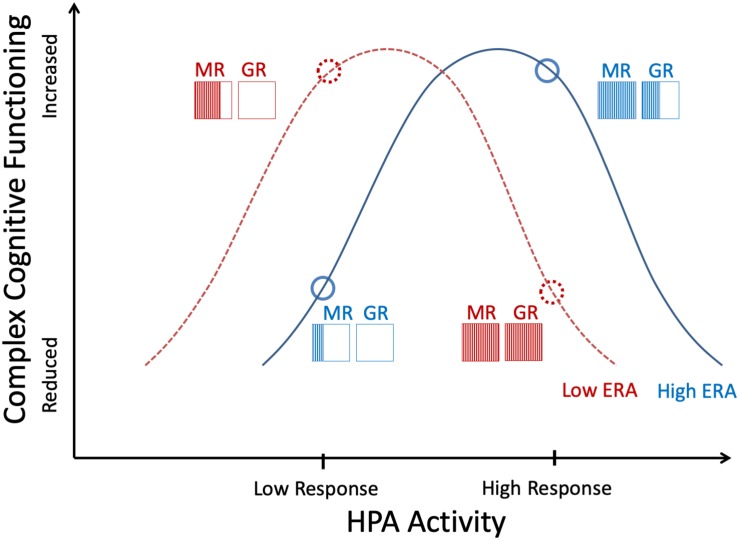
Hypothesized ERA × cortisol effects on performance, based on the hormetic model by Lupien et al. (2005). MR = mineralocorticoid receptors, GR = glucocorticoid receptors. The four points indicate the hypothesized disordinal interaction between cortisol response and ERA in predicting intuitive cognition.

Individual differences in ERA might thus function as an individual-differences factor that plays a pivotal role in the relationship between cortisol release and cognitive performance. Studies showed that glucocorticoid receptors in the prefrontal cortex play a role in emotion and stress regulation (Diorio et al., 1993; McKlveen et al., 2013). The hypothesized relationship might be described as follows and as depicted by the graph in Figure 1: Differing stress reactivity levels in low versus high ERA individuals may be represented by different cortisol receptor quantities within the prefrontal cortex which has shown to play a crucial role in emotion regulation (Quirk and Beer, 2006). The different amount of GR in the prefrontal cortex of individuals with varuous ERA is only hypothesized. However, if we follow this plausible suggestion, a stress situation should mainly impact higher glucocorticoid receptor quantities in low ERA individuals but lower glucocorticoid receptor quantities in high ERA individuals, reflecting an interaction effect of cortisol and ERA on cognitive performance.

An important cognitive function that is likely to be influenced by ERA and stress is intuitive thought, in terms of the “capacity for direct knowledge, for immediate insight without observation or reason” (Myers, 2004, p. 1). Here we investigated the degree to which individual differences in ERA in interaction with the cortisol response to a public speaking scenario and subsequent reminders thereof predict intuitive cognitive processing as measured by accurate judgments about semantic coherence (Mednick, 1962).

Investigating the relationship between negative affect and intuition, previous research has led to inconsistent results (for a review see Baas et al., 2008). For example, Vosburg (1998) and Bolte et al. (2003) demonstrated that negative mood impairs divergent thinking and performance in a coherence judgments task, whereas others (e.g. Adaman and Blaney, 1995; for a review see Baas et al., 2008) found the opposite. In a meta-analysis that included a semantic coherence task as one among other kinds of creativity tasks (Baas et al., 2008) it was shown that rather than hedonic tone (positive vs. negative mood) or mere activation (deactivating vs. activating mood) it is the mood-associated regulatory focus (promotion vs. prevention focus) that determines the mood-creativity relationship. That is, deactivating states have little to no impact on creativity (e.g. sadness, relaxed), while activating promotion-focused states (e.g. happiness) enhance creativity and activating prevention-focused states (e.g. fear) impede creativity. Investigating the link to emotion regulation, Baumann and Kuhl (2002) concluded from their study that high ERA (action orientation) increase the ability to access semantic networks under negative affect and therefore improves the ability to make correct coherence judgments. By contrast, low ERA individuals showed a decrease in coherence judgment performance. In similar vein, in individuals with a major depression, rumination levels moderated the adverse impact of negative affect on accurate semantic coherence judgments in a sample of individuals with depression (Remmers et al., 2015; Remmers et al., 2017). This finding is of particular relevance here because rumination, in its non-clinical variant, is a typical phenomenon in individuals with low ERA (Kuhl and Beckmann, 1994).

Interestingly, overlaps seem to exist for brain areas involved in intuition and areas with a high density of glucocorticoid and mineralocorticoid receptors. That is, on the one hand, brain areas involved in intuition and insight related tasks are found in the right hemisphere (Beeman et al., 1994; Bowden and Beeman, 1998; Beeman and Bowden, 2000; Bowden and Jung-Beeman, 2003; Jung-Beeman et al., 2004; Bowden et al., 2005; Jung-Beeman, 2005; Goldstein et al., 2010). On the other hand, during the coherence judgment task, there is also (pre)frontal activation including areas associated with top-down processing and working memory (for reviews see Dietrich and Kanso, 2010; and Mihov et al., 2010). For example, in a TMS study, anodal (positive) stimulation of the left dorsal prefrontal cortex improved performance in the coherence judgment task (Cerruti and Schlaug, 2009). Due to this overlap of areas involved in intuition tasks and areas with a high density of corticoid receptors, it is very likely that there is a direct relationship between the amount of circulating cortisol and performance in tasks like the Remote Associates Test (RAT; Mednick, 1962). However, to our knowledge there has only been one study investigating a similar relationship. In this study, acute stress impaired performance in the RAT task, but administering propranolol, a beta-adrenergic antagonist, tended to block this effect (Alexander et al., 2007).

We investigated the moderating impact of ERA on the relationship between HPA-related stress and intuition by inducing social-evaluative stress and reminding participants thereof later. Stress-reminders were words reminding of the stress task that preceded half of the RAT triads presented after stress induction. We measured saliva cortisol concentration and the performance in a semantic coherence task, while controlling for performance after neutral prime words preceding the other half of the RAT triads. Based on the hypothesized relationships depicted by Figure 1, we predict a two-way interaction of ERA and cortisol change. That is, high ERA individuals with an increase in cortisol levels would show an enhancement in correct coherence judgments, while low ERA individuals with a comparable increase in cortisol levels would show no change or even a decline in performance. However, low ERA individuals with low cortisol changes should show similar or even better (Koole et al., 2012) performance than high ERA individuals with comparably low cortisol change.

## Method

### Participants and Design

Forty-nine right-handed students (32 female) from Osnabrück University, Germany, all Caucasian, aged 18 to 33 years (*M* = 22.48, *SD* = 3.33), participated for 15 Euro or course credit. They all gave oral informed consent before participating in the study and reported to have no psychiatric history or taking any medication. The study conformed to Declaration of Helsinki principles. The design of the study included the factors ERA (continuous, between participants), cortisol change in response to the stress induction (continuous, between participants), and prime type (neutral vs. stress-reminding, within-participants). We measured performance in a semantic coherence task as a dependent variable.

### Materials

#### Stress Induction

Stress was induced by a variant of the well-established and standardized Trier Social Stress Test (TSST; Kirschbaum et al., 1993), which is a reliable method to induce stress in a laboratory setting and has been shown to increase cortisol concentration (Dickerson and Kemeny, 2004). Specifically, after having arrived, participants were informed about their task to deliver a free speech in order to convince an expert group (which would not be present but would look at the video recordings afterward) that they were the perfect applicant for a vacant position. After a preparation period of 5 min, a speech of 5 min followed and afterward participants were asked to do challenging mental arithmetics for 5 min. Accordingly, stress is likely to derive from individuals’ fear of social evaluation as a potential reaction to failure. During the mock simulated job interview and mental arithmetic task, participants’ speeches were recorded with a video camera and they were told that their video will later be analyzed and evaluated by an expert group. This ensured that moderate rather than intense stress was induced in a way that differences in personality can have a stronger influence on cortisol release.

#### Remote Associates Test

To assess intuition we applied a coherence judgments task, the RAT (Mednick, 1962), where participants have to indicate whether a word triad presented to them is semantically coherent, in the sense that they have a feeling that there exists a fourth word which is a low associate to all the three presented words (e.g. for *goat*, *pass*, *green*, the low associate *mountain* exists), or not (e.g. *spoon*, *lion*, *ticket*). The RAT stimulus set and variations thereof have been used in studies investigating creative processes and convergent thinking, such as insight, and intuition. In particular, it has already been used to examine the relationship between affective states and intuition (Bolte et al., 2003) and personality differences (Baumann and Kuhl, 2002). As stimuli, we took a German RAT version that was based on the triads from a study from Bowers et al. (1990). Half of the triads were coherent, such that a fourth word exists, which is a low associate of the three presented words. The other half of the triads were incoherent, such that there exists no common fourth word. Participants were presented with 136 word triads in a pseudo-randomized order.

Each RAT trial started with presenting a fixation cross for 500 ms, followed by the prime word for 300 ms, followed by the target word triad remaining on screen upon decision. Participants indicated by bimanual key press whether the presented words had a fourth solution word being related to the three presented words or not. They were instructed that they had infinite time but that they should react as soon as they had a feeling whether the presented word triad was coherent or incoherent. 2500 ms after button press the next trial started. Retrieval of the solution word was not included, but participants were instructed to intuitively judge the triads, no matter whether they had a particular solution word in mind or not. This assures that holistic thinking is tested independently of conscious processes (cf. Ilg et al., 2007; McCrea, 2010; Zhang et al., 2016).

#### Priming

During half of the triads, neutral primes (e.g. *tree, soap, plate*) were shown. During the other half, TSST-related and therefore stress-reminding primes (e.g. *failure*, *doubt, shame*) were shown. All prime words were taken from the Berlin Affective Word List Reloaded (Võ et al., 2009). Four ANOVAs with two factors each were conducted. Each one of the four ANOVAs dealt with one word characteristic, that is, we conducted one ANOVA with valence as dependent variable, one with arousal, one with number of syllables, and one with frequency per one million words as dependent variable. Each one of the ANOVAs had two factors, that is, prime type (neutral vs. stress-reminding) and coherence of the following word triad (coherent vs. incoherent). These four ANOVAs were calculated to make sure whether prime characteristics (that is, valence, arousal, syllables, frequency) did or did not differ between negative/neutral and coherent/incoherent trials. Neutral versus stress-reminding primes differed significantly in valence, *p* < 0.001, and arousal, *p* < 0.001, but not in syllables and word frequency, *p*s > 0.12. Prime characteristics did not differ in coherent versus incoherent trials, all *p*s > 0.24, and there were no significant interaction effects of prime type and coherence type on the word properties, all *p*s > 0.30.

#### Measuring Emotion Regulation Abilities

To measure ERA we applied a 12-items subscale of the Action Control Scale (Kuhl and Beckmann, 1994), action orientation after failure. Each item describes a particular situation and participants are asked to indicate which of the two given alternatives describes best the way they would react. An example for a situation is *If I’ve worked for weeks on one project and then everything goes completely wrong with the project.* One response alternative describes an action-oriented approach toward the problem (here *it bothers me for a while, but then I don’t think about it anymore*), whereas the other alternative describes a state-oriented approach (here *it takes me a long time to adjust myself to it*). The final score captures the number of action-oriented responses. Action orientation after failure is particularly suited to measure ERA in the present context as we induced social evaluative stress (typically deriving from fear of failure) and additionally used stress-reminding priming.

### Experiment Procedure and Cortisol Assessment

At home, participants completed a battery of questionnaires, including the Action Control Scale, to measure ERA. They were asked not to eat and drink anything 2 h before coming to the laboratory. All experimental sessions started between 1200 and 1500 h and lasted for approximately 2.5 h. After having arrived in the laboratory, participants were seated in a comfortable chair and an EEG cap was applied. Because the present findings are unrelated to EEG findings, details and method of the EEG procedure will not be reported here. After EEG baseline measurement and associated tasks that were unrelated to the RAT task or any kind of holistic or intuitive thinking, a cortisol baseline sample was taken (T1). Afterward, participants engaged in the TSST and afterward took the second cortisol sample (T2 ∼ 20 min after the stressor onset). After having been seated in front of the computer and another EEG measurement, a third cortisol sample (T3 ∼ 45 min after the stressor onset) was taken. Afterward, participants read task instructions on the screen and completed training trials before the 136 trials of the RAT started. In the end, a fourth cortisol sample was taken (T4 ∼ 60 min after the stressor onset). In order to collect the saliva samples, participants were instructed to chew on the salivette for 45 s. The samples were kept at −20°C until they were sent via personal courier and stored in a cooling box to the biochemical laboratory at the University of Trier to be analyzed by a time-resolved immunoassay with fluorescence detection (Dressendörfer et al., 1992). The intra-assay coefficient of variation was between 4.0 and 6.7%, whereas the corresponding inter-assay coefficients of variation were between 7.1 and −9.0%. The lower detection limit was 0.43 nM.

In the original TSST study (Kirschbaum et al., 1993) and a review of the protocol after 10 years of research (Kudielka et al., 2007), saliva cortisol is said to peak at 30 to 40 min after the onset of the TSST, that is 10 to 20 min after cessation of the stress task. Therefore we expected our cortisol peak at the third measurement, which took place 45 min after the stress onset, that is, 25 min after cessation of the stress task.

### Statistical Analyses

For each participant, as a measure of cortisol levels change, we calculated the area under the curve with respect to increase (AUCi) using the four time points of cortisol saliva measurement (Pruessner et al., 2003). For each participant, a discrimination index A’ was calculated for the performance after stress-reminding and after neutral primes separately. A’ is a non-parametric alternative to d’, based on participants’ hit and false alarm rates, can be calculated even with a small number of stimuli, or when hit rates of 1 or false alarms of 0 occur, and is highly correlated with d’ (Pollack, 1970). An A’ index of 1.0 refers to perfect discrimination, whereas 0.5 refers to chance performance. As we used a mild variant of the TSST by recording videos for the participants, we expect individuals to be at the beginning of the inverted-u shape, as indicated in Figure 1. Therefore, we modeled a linear relationship in our analysis. We used a blockwise multiple regression to investigate the moderating effect of the continous ERA variable on the relationship between continuous A’ scores after stress reminding prime words and cortisol levels, measured by the continuous AUCi (see Table 1). Average A’ scores after stress reminding prime words were regressed on performance after neutral prime words (entered in block 1), ERA and AUCi (entered in block 2) and the ERA × AUCi interaction (block 3). For the ease of interpreting and displaying effects and to reduce non-essential collinearity (West et al., 1996; Hayes, 2017), all first order predictors were centered at their individual means.

**TABLE 1:** Blockwise regression analysis summary for predicting performance (A’) after stress-reminding prime words.

	Unstandardized regression coefficient				Explained variance		
			
Variable	*B*	95% CI	*P*	β	*R*^2^	Δ*R*^2^	*p*
Step 1					0.23		
**Constant**	**0.79**	**[0.76, 0.81]**	**(<0.001)**				
**After neutral prime words**	**0.45**	**[0.21, 0.69]**	**(0.001)**	**0.48**			
Step 2					0.23	0.00	(0.949)
**Constant**	**0.79**	**[0.76, 0.81]**	**(<0.001)**				
**After neutral prime words**	**0.45**	**[0.20, 0.70]**	**(0.001)**	**0.48**			
ERA	−0.15 × 10^–3^	[−0.01, 0.01]	(0.748)	0.01			
AUCi	−0.02 × 10^–3^	[−0.11 × 10^–3^, 0.08 × 10^–3^]	(0.972)	−0.04			
**Step 3**					**0.39**	**0.17**	**(0.001)**
**Constant**	**0.78**	**[0.76, 0.81]**	**(<0.001)**				
**After neutral prime words**	**0.37**	**[0.14, 0.60]**	**(0.002)**	**0.39**			
ERA	−0.38 × 10^–3^	[−0.01, 0.01]	(0.922)	0.01			
AUCi	−0.02 × 10^–3^	[−0.10 × 10^–3^, 0.07 × 10^–3^]	(0.709)	−0.04			
**ERA × AUCi**	**0.06 × 10**^–^**^3^**	**[0.03 × 10**^–^**^3^, 0.09 × 10**^–^**^3^]**	**(0.001)**	**0.42**			

*CI = confidence interval. AUCi = Area under the curve with respect to increase. ERA = emotion regulation abilities. Rows with effects *p* < 0.05 are highlighted in bold.*

Additionally to the frequentist approach, we also calculated a Bayesian regression and estimated Bayes Factors (BF) for the models. This has several advantages as compared to the frequentist approach: It needs smaller sample sizes (Rouder and Morey, 2012; van de Schoot and Depaoli, 2014), is more conservative (Stanton, 2017), and allows an estimation of the credibility of the model and the increase of credibility of one model versus another model (Jeffreys, 1998; Dienes, 2014; Dienes and Mclatchie, 2017). Posterior distributions were calculated using the Markov Chain Monte Carlo (MCMC) method with 10,000 iterations, based on mixed g-priors, which are suitable for estimating the null hypothesis of a regression model (Liang et al., 2008; Rouder and Morey, 2012). Resulting 95% highest density intervals (HDI; Kruschke, 2015) indicate the most credible values for the population parameters of interest.

## Results

### Descriptives

Emotion regulation abilities (*M* = 4.62, *SD* = 2.86, range: 0–12, Cronbach’s α = 0.73) was uncorrelated with cortisol level at the first (baseline) measurement (*M* = 7.60 nmol/l, *SD* = 5.54, range: 2.19–28.38), at the second measurement (*M* = 8.99 nmol/l, *SD* = 4.56, range: 4.28–21.76), at the third (post stress) measurement (*M* = 10.54 nmol/l, *SD* = 6.67, range: 3.54–33.96), at the fourth measurement (*M* = 9.24 nmol/l, *SD* = 5.77, range: 3.64–32.45) and the difference between the first and third measurement (*M* = 2.94 nmol/l, *SD* = 6.38, range: −12.32–24.88), all *p*s > 0.30. The increase of cortisol level over the course of time was significant, that is, cortisol level increased from first (baseline) to second measurement, *t*(48) = 2.344, *p* = 0.023, increased from second to third (post stress) measurement, *t*(48) = 3.159, *p* = 0.003, and decreased from third (post stress) to fourth measurement, *t*(48) = −5.101, *p* < 0.001. ERA was uncorrelated with the AUCi (*M* = 105.71, *SD* = 268.12, range: −684.75–1023.53), *p* = 0.774.

**FIGURE 2:**
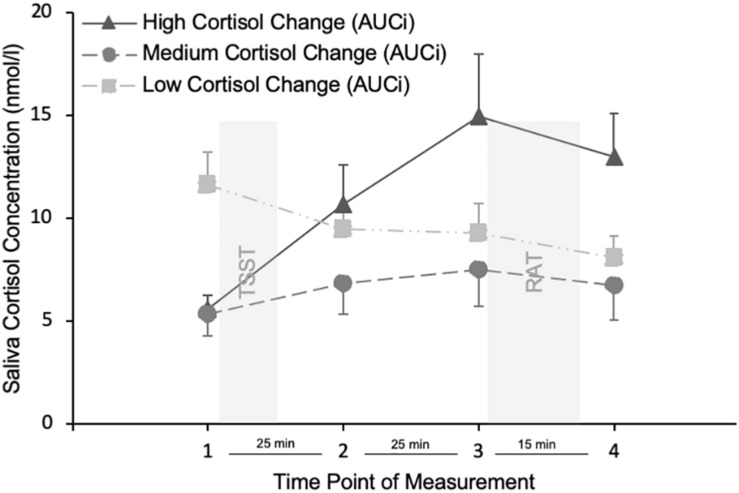
Cortisol concentration at the four time points of measurement. Only for displaying reasons, participants were divided into low medium and high cortisol change groups, based on their AUCi values. TSST = stress induction using the Trier Social Stress Test. RAT = holistic processing task using the Remote Associates Test. Error bars indicate the standard error of the mean.

For reasons of graphical illustration we calculated a difference score, that is, the cortisol change from the first to the third measurement and splitted participants into three groups. Cortisol remained a continuous variable in all analyses. Figure 2 shows that in responders (high cortisol change group), stress induction was associated with an increase in cortisol from the first to the third measurement and that they show a peak cortisol concentration at the third measurement. A proxy of cortisol changes above 1.5 nmol/l can be used to identify responders (Miller et al., 2013). Although, only 57.1% of participants were responders with a cortisol change of more than 1.5 nmol/l, with a mean cortisol change of 2.94 nmol/l from the first to the third measurement, our average participant can be regarded as a responder and with the high cortisol change group peaking at 14.94 nmol/l they were in the range of previous TSST studies’ saliva cortisol response (Kirschbaum et al., 1993).

### RAT Performance

Out of the 68 coherent (68 incoherent) triads, participants judged 37.31 (49.24), *SD* = 8.81 (9.18) triads correctly. On average, participants judged 63.64% (*SD* = 5.84%) of the word triads correctly. Participants of this study had an average A’ of 0.72 (*SD* = 0.08).

The blockwise regression model for average A’ scores after stress reminding prime word was regressed on performance after neutral prime words (entered in step 1), ERA and AUCi (entered in step 2) and the ERA × AUCi interaction (step 3) is associated with an explained variance of *R*^2^ = 39.3% where the ERA × AUCi interaction accounts for an Δ*R*^2^ = 16.54% and shows a partial correlation of *r* = 0.463, 95% CI [0.20, 0.66], *p* = 0.001, corresponding to an effect size of *f*^2^ = 0.27. For detailed statistics see Table 1. Identifying influential cases showed that for one person, Cook’s distance was >1. If analyses are repeated without this influential case, the two-way interaction of interest is insignificant, *p* > 10. We present additional analyses and interpretation concerning outliers in our data in the Supplementary Material. We found a corresponding BF for the full model (neutPrimes + ERA + AUCi + ERA × AUCi) as compared to the null model of *BF* = 196.33. The comparison of the full model against the model without the interaction (neutPrimes + ERA + AUCi) showed a *BF* = 35.91, indicating very strong evidence for H_1_ hypothesis according to Jeffreys (1998). Analysis of the posterior distribution via MCMC estimated a mean *R*^2^ of 0.31 with a 95% HDI [−0.04 0.55] and an estimated unstandardized regression coefficient for the interaction *b* = 5.11^∗^10^–5^ (SD = 1.29^∗^10^–5^) and a 95% HDI [1.85^∗^10^–5^ 8.45^∗^10^–5^].

**FIGURE 3:**
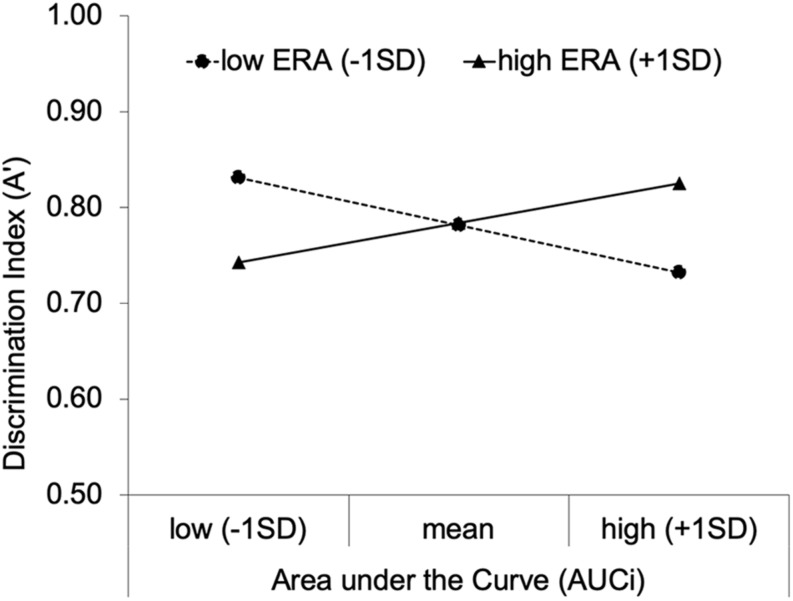
Regression Slopes for the moderating effect of emotion regulation abilities on the relationship between saliva cortisol change (Area under the Curve; AUCi) and the performance in the RAT after negative primes (discrimination index; A’).

To disentangle this interaction, we conducted simple slopes analyses controlling for mean performance after neutral primes. Among low ERA individuals (1SD below the mean), AUCi had a negative effect on A’, *B* = −0.19 × 10^–3^, 95% CI [−0.31 × 10^–3^, −0.06 × 10^–3^], β = −0.54, *p* = 0.006, and among high ERA individuals (1SD above the mean), AUCi had a positive effect on A’, *B* = 0.15 × 10^–3^, 95% CI [0.03 × 10^–3^, 0.28 × 10^–3^], β = 0.45, *p* = 0.019. To give an example, for low ERA individuals, an AUCi increase from mean to 1SD above the mean is associated with A’ after negative primes decreasing from 0.78 to 0.73. This decline in A’ for example, represents an increase in the false alarm rate from 11 to 18% at a constant hit rate of 90%, or, for example, a decrease in the hit rate from 89 to 82% at a constant false alarm rate of 10%. Looking at the effects from another perspective, the effect of ERA on A’ at low levels (1 SD below the mean) of AUCi was significant, *B* = −0.02, 95% CI [−0.03, 0.00], β = −0.48, *p* = 0.012, as well as the effect of ERA on A’ at high levels (1SD above the mean) of AUCi, *B* = 0.02, 95% CI [0.00, 0.03], β = 0.50, *p* = 0.010. For a visualization of the results based on simple slope analyses, see Figure 3.

## Discussion

The present research investigated effects of ERA on intuition as moderated by cortisol responses to the TSST and subsequent stress-task reminders. As expected, we found a disordinal (cross-over) interaction: Cortisol increases were associated with higher performance in high ERA individuals but with lower performance in low ERA individuals, and low or absent cortisol increases were associated with lower performance in low ERA individuals but with higher performance in high ERA individuals.

Cortisol change and ERA interacted to predict the facilitating effect of stress-reminding primes on coherence judgments. For individuals who showed a small increase in cortisol, the TSST may have been less of a challenge, and low ERA was associated with the largest prime-induced increase in performance. By contrast, for individuals who showed a large increase in cortisol, the TSST may have been a challenge, and high ERA was associated with the largest prime-induced increase in performance. Increased performance of low ERA individuals at low compared to high challenge levels may be related to their inclination to show already high levels of sympathetic activation in relatively low-stress conditions involving negative stimuli (Kuhl, 2000; Tops et al., 2015). Because these individuals easily show high levels of sympathetic activation, they sooner (i.e. at a lower level of challenge) than high ERA individuals reach the threshold of a protective inhibitory mechanism that protects them against sympathetic over-activation and dampens the appraisal process (Tops et al., 2015).

Our finding that ERA moderates the effect of cortisol on cognitive functioning is compatible with more general findings of socio-cognitive processes working differently for individuals in both clinical or non-clinical affective conditions. For example, it has been found that social exclusion is not equally painful and inclusion not equally pleasant for different individuals (Seidel et al., 2013). Furthermore, it has been shown that affective manipulations such as fear conditioning do not necessarily show adverse effects on cognitive performance but that trait anxiety plays a pivotal role (Fox et al., 2012). Our finding along with these findings clearly demonstrate the relevance of individual differences in whether situational affordances or stressors influence performance and in which way.

As a consequence of inhibited appraisal frustrating the processing cycle, at higher stress levels individuals with low ERA less easily shift via the analytic system activation toward the activation of the system of internal models and self-schemas, decreasing performance in the RAT. By contrast, in low-stress conditions high ERA individuals typically show lower levels of appraisal and sympathetic responses to negative stimuli. Hence, they have more space for increases in sympathetic arousal with increasing levels of stress and demand and in response to negative stimuli before they reach their threshold of protective inhibition. This different positioning of low and high ERA individuals on the inverted-U relationship (Figure 1) between level of demand or tonic arousal (as indexed by cortisol response) and appraisal responses to negative stimuli may explain why, after stress-reminding primes, low ERA individuals outperformed the high ERA individuals on the RAT if they showed a low cortisol change but the high ERA individuals outperformed the low ERA individuals if they showed a high cortisol change. Evidence suggests that individual differences in mineralocorticoid (Vogel et al., 2016) and/or glucocorticoid (Lupien et al., 2005) receptors quantity and/or quality underlie working memory and prefrontal cortex activity which are both related to individual differences in emotion regulation abilities (Kuhl, 2001). These results show that effects of HPA-related stress on cognitive performance need to consider differences in emotion regulation and future research will need to test our interpretation and hypotheses about underlying processes.

Interactions between stress and ERA have not only been related to intuitive thought but also to other processes (Kuhl et al., 2015). For example, ERA are associated with a better differentiation between own goals and other individuals’ expectations (Baumann and Kuhl, 2003), with increased intuitive self-awareness (Kazén et al., 2003), and with an increased congruence between needs and goals (Baumann et al., 2005). It has been argued (Kuhl, 2000; Koole et al., 2012; Kuhl et al., 2015; Quirin et al., 2015) and empirically supported (Quirin et al., 2011a; Quirin et al., 2014) that high ERA individuals capitalize on the activation of a neuropsychological system that may be called “extension memory” because of its ability to process remote associations (Kuhl, 2000; Tops et al., 2014a, b; Engel and Kuhl, 2015; Kuhl et al., 2015 for similar conceptualizations), which is beneficial for successfully solving triads in the RAT (Jung-Beeman et al., 2004). This ability is particularly useful for sustainably (rather than defensively) coping with stress as it enables a person to put negative experiences in perspective and to adopt a mindful (broad) monitoring of internal conditions such as needs, emotions, and sensations. Therefore, usage of extension memory might constitute an important variable that might moderate the present effects of social stress and concurrent cortisol release on holistic processing.

The current study focused on holistic but not analytical processing. We would expect different effects on tasks requiring predominantly analytical processing or a narrow scope of attention. Specifically and in line with previous theorizing (e.g. Kuhl, 2000), individuals with low ERA may show increased rather than reduced performance under stress in analytical tasks such as word-spelling correction (Kazén et al., 2015). However, future studies should directly test the assumption that the present effects are specific for holistic processing but do not apply to analytical processing.

As a limitation, we need to stress that, although our findings were in line with our hypotheses, the significant effect was driven by two statistical outliers with respect to cortisol changes. These cases were still within a usual range of cortisol change, and therefore we decided not to remove them. Rather, we presented analyses with and without them in the Supplementary Material. This leads us to say that the data support our hypothesis partially but not fully, and that future research is needed to replicate our findings. Moreover, the menstrual cycle phase influences the cortisol response to laboratory psychosocial stress (Montero-López et al., 2018). As our interest was in ERA rather than gender differences, we did not screen for women’s physiological cycles. Not least, we did not include a non-stress group that might have controlled for effects of the TSST. These limitations might be considered in future studies.

## Conclusion

The relationships between neuroendocrine functioning and different types of cognitive processing are more than complex, which has probably contributed to an absence or inconsistency in findings during the last two decades or so. The present work was conducted to shed more light on this complexity by adopting an individual differences approach. The personality variable ERA moderated the relationship between cortisol response and holistic cognitive processing in a way that no relationship was found on the level of the average sample. Therefore, it is necessary to consider personality, ERA in particular, in stress research. The proposed u-shape model of differential HPA functioning might be helpful for future research on individual differences in the relationship between stress and cognitive performance.

## Data Availability Statement

The datasets generated for this study are available on request to the corresponding author.

## Ethics Statement

The studies involving human participants were reviewed and approved by the Ethics Commission of the Osnabrück University. The patients/participants provided their written informed consent to participate in this study.

## Author Contributions

RD, JK, and MQ developed the concept and experimental design for the study. RD performed the measurements. ER analyzed the data. MQ and RD supervised the analyses. ER, RD, JK, MT, and MQ interpreted the results. ER took the lead in writing the manuscript, and RD, JK, MT, and MQ were involved in its revision.

## Conflict of Interest

The authors declare that the research was conducted in the absence of any commercial or financial relationships that could be construed as a potential conflict of interest.
