# Supplementary material for: Personality, Stress, and Intuition: Emotion Regulation Abilities Moderate the Effect of Stress-Dependent Cortisol Increase on Coherence Judgments
Source: Front Psychol. 2020 Feb 27;11:339. doi: 10.3389/fpsyg.2020.00339 (PMC7057143; doi:10.3389/fpsyg.2020.00339)
Supplement: Supplementary file 1 [file Data_Sheet_1.PDF]

Supplementary Material

1 Supplementary Data

As suggested by one of the reviewers, we elaborate on our outliers in this supplementary material. We believe, as explained in the next paragraph, that our outliers are rare cases but not outliers in a narrow sense. We provide more specific information about the outliers below, in order to not confront the reader with too many statistical details in the main article.

We used the term "outlier" to refer to different statistical parameters (e.g. Cook's distance, residuals etc.). In our study, one participant was an "influential case" (large Cook's distance, which we reported and had removed) and another participant had a relative high residual (who we kept in our original analysis). Both were unusual because of their values on the AUCi variable. But when inspecting the original cortisol measures (t1-t4), both showed values in a usual range given the literature. The former case showed a quite low cortisol level upon arrival and had a large increase with respect to the TSST intervention. The latter participant showed a pattern vice versa: high cortisol level upon arrival but a quite low reaction to the TSST, both resulting in unusually appearing AUCi values. As their original values were in reasonable ranges of the population of interest, we kept them in the analyses but to report this issue in the article.

In Figure 1, we present the partial regression plots for the interaction terms for 49, 48, and 47 participants, respectively.

In Figure 2-4, we present the results of a bootstrap procedure to estimate the median of the regression coefficients and the 95% bca confidence intervals with and without 1 and 2 "outliers". As can be seen, with all participants included (**Figure 2**) the robust bootstrap estimation shows a bimodal distribution for the regression coefficients for the interaction, but with lower and upper bounds larger than zero, indicating a "significant effect". After removal of the "outliers" **(Figure 3 & Figure 4)** the effect is attenuated, but still positive.

To get an impression of the effect of the outlier removal, we can calculate an example. If we take the estimated regression coefficients from the bootstrap procedure for low ERA individuals (-1SD), an increase in AUCi from mean to 1 SD above the mean is associated with A' after negative primes decreasing from median 0.787 to 0.740, 95% CI [0.774 to 0.734, 0.777 to 0.723]. The bootstrapped median decline in A' from 0.787 to 0.740, represents, for example, an increase in the false alarm rate from 10.6% to 16.5% at a constant hit rate of 90%. After removing the two outliers, this decline changes to median A' decreasing from 0.797 to 0.782, 95% CI [0.796 to 0.779, 0.798 to 0.779]. To have an indicator for the lower bound of the effect, illustrating the effect at the lower bound of the confidence interval without the two "outliers", that is, a decline in A' from 0.796 to 0.779, for example, represents an increase in the false alarm rate from 9.5% to 11.5% at a constant hit rate of 90%.

Because frequentistic decision making heavily relies on significance [which is not very informative and should not solely been used for decision making, according to the APA (2010), the Task Force on Statistical Inference (Wilkinson and the Task Force on Statistical Inference, 1999)] or the

American Educational Research Association (AERA, 2006), which in turn is heavily influenced by sample size, we decided to conduct bayesian analyses. The results of the MCMC method for the analyses with and without the “outliers” can be found in **Figures 5-7**, which show the HDI for the regression coefficients of the interaction terms, as well as the overall R^2 of the models. The HDI must not be interpreted in terms of frequentistic confidence intervals! After application of the MCMC algorithm, the HDI shows the most credible values for the parameters of interest, which in turn should not be used for a dichotomous decision of “significant” vs. “not significant”. All three models come to similar conclusions, which are also in line with the bootstrap procedure, i.e. with and without potential outliers, parameter estimation reliably show positive values for the regression coefficients of the interaction terms. For the estimation of R^2, it can be seen that in the original sample some values of the 95% HDI fall below 0, but the distribution has a negative skew, i.e. the mass of the distribution is concentrated on the right of the figure.

2 Supplementary Figures and Tables

(A)

(B)

(C)

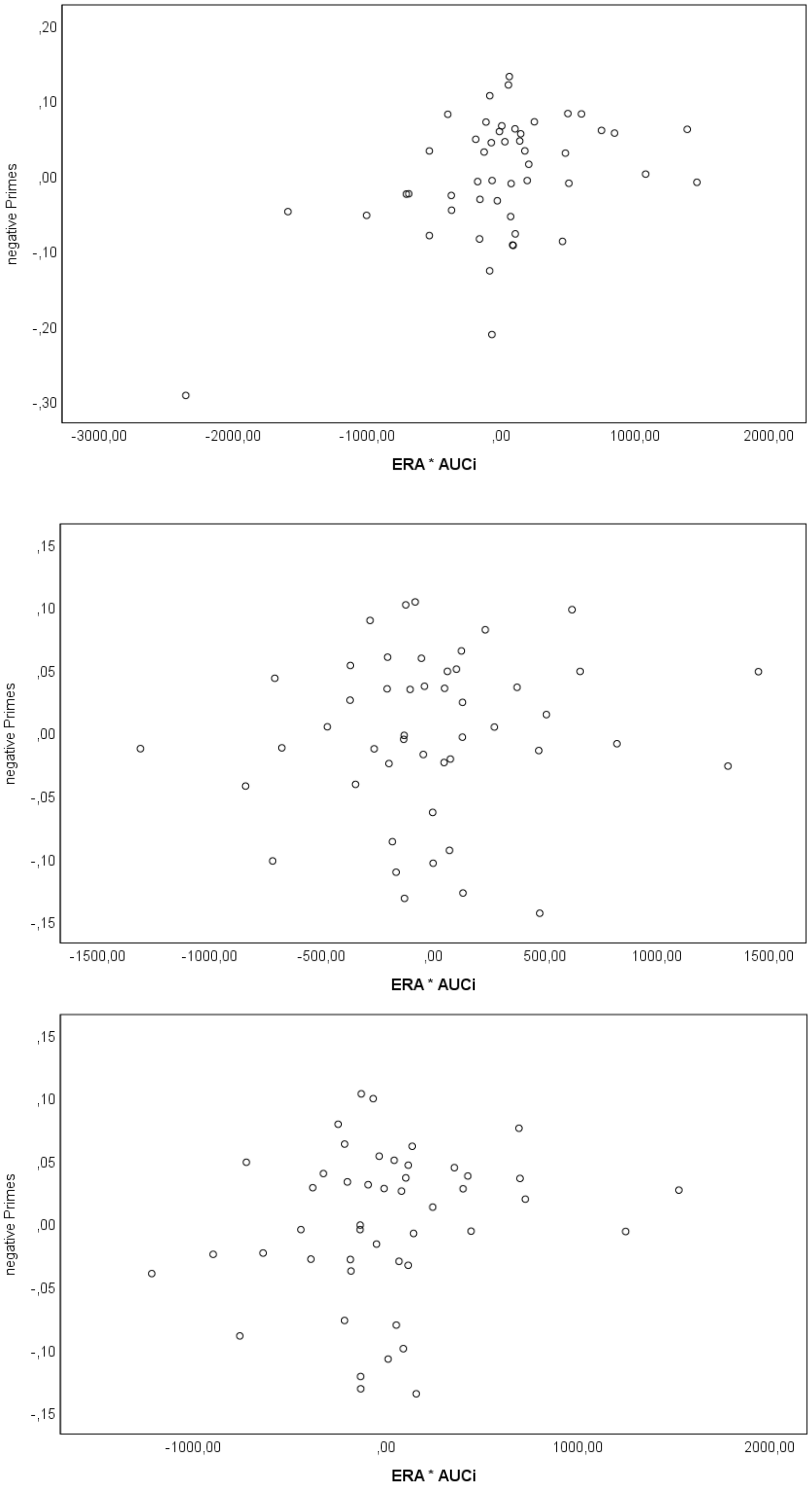

Supplementary Figure 1. Partial Regression plots with (A) 49, (B) 48 (removal of one potential outlier), and (C) 47 participants (removal of two potential outliers).

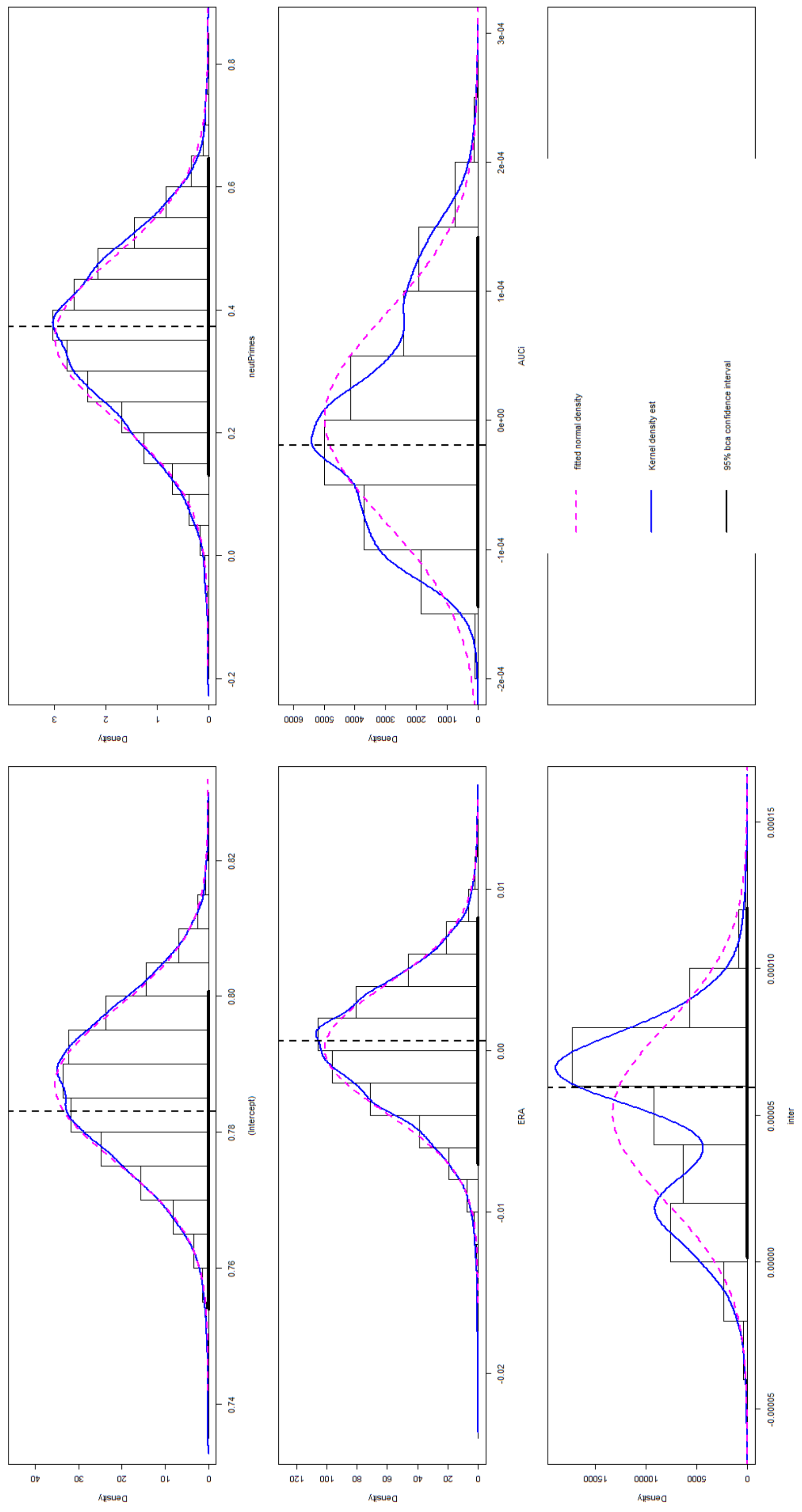

Supplementary Figure 2. Bootstrap results for regression coefficients with 49 participants

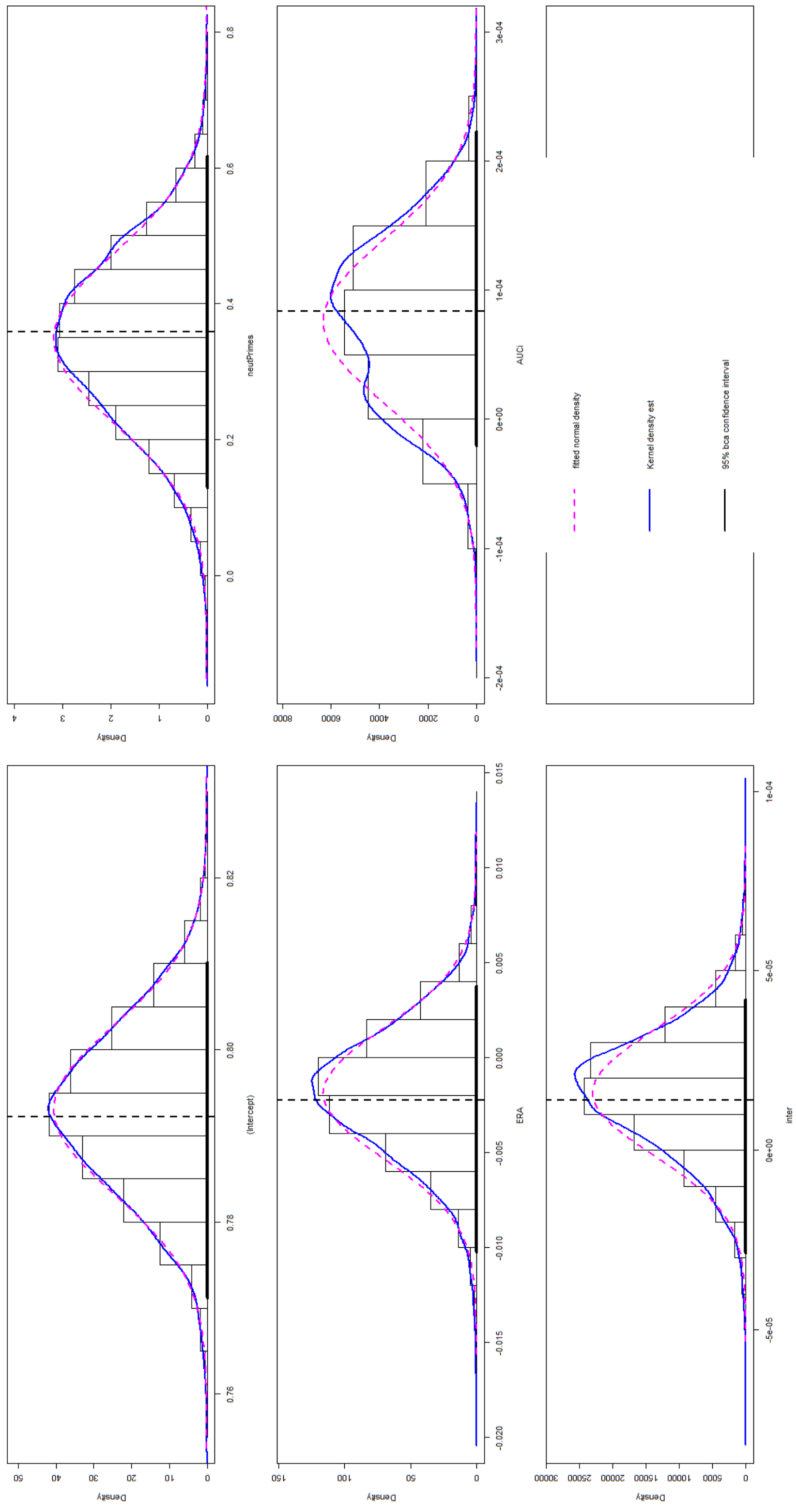

Supplementary Figure 3. Bootstrap results for regression coefficients with 48 participants (removal of one potential outlier)

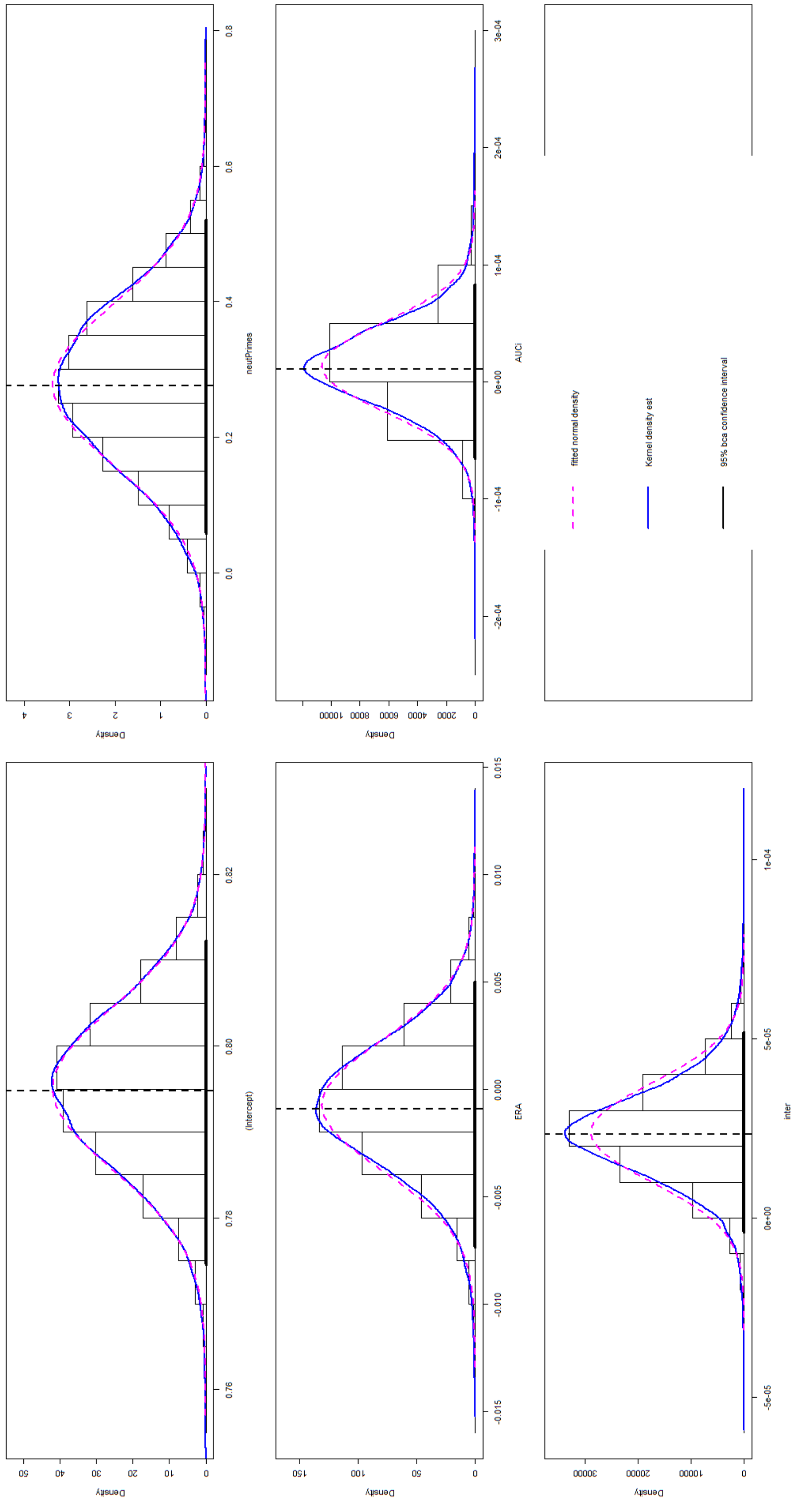

Supplementary Figure 4. Bootstrap results for regression coefficients with 47 participants (removal of two potential outliers)

(A)

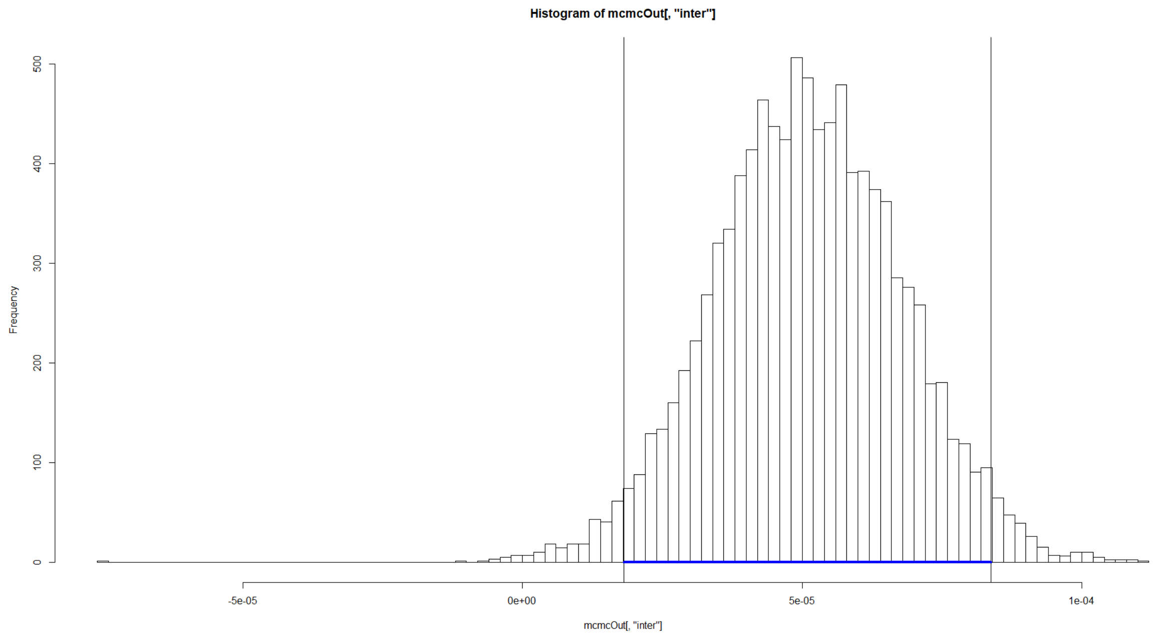

(B)

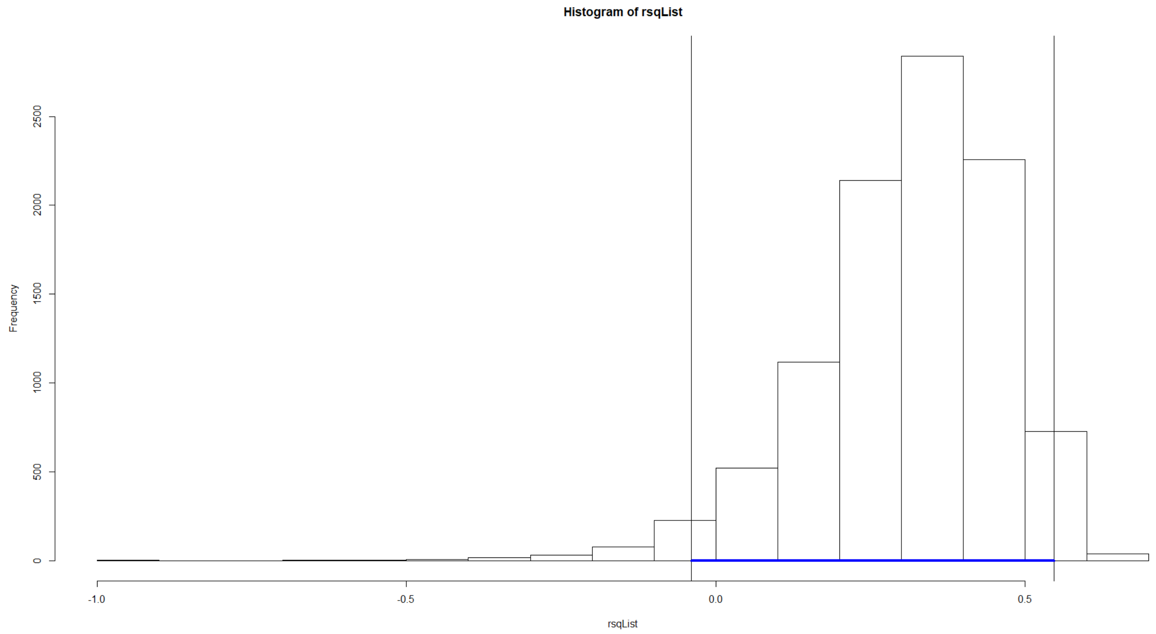

Supplementary Figure 5. Bayesian Analysis with 49 participants. (A) 95% HDI for the interaction regression coefficients. (B) 95% HDI for R^2.

(A)

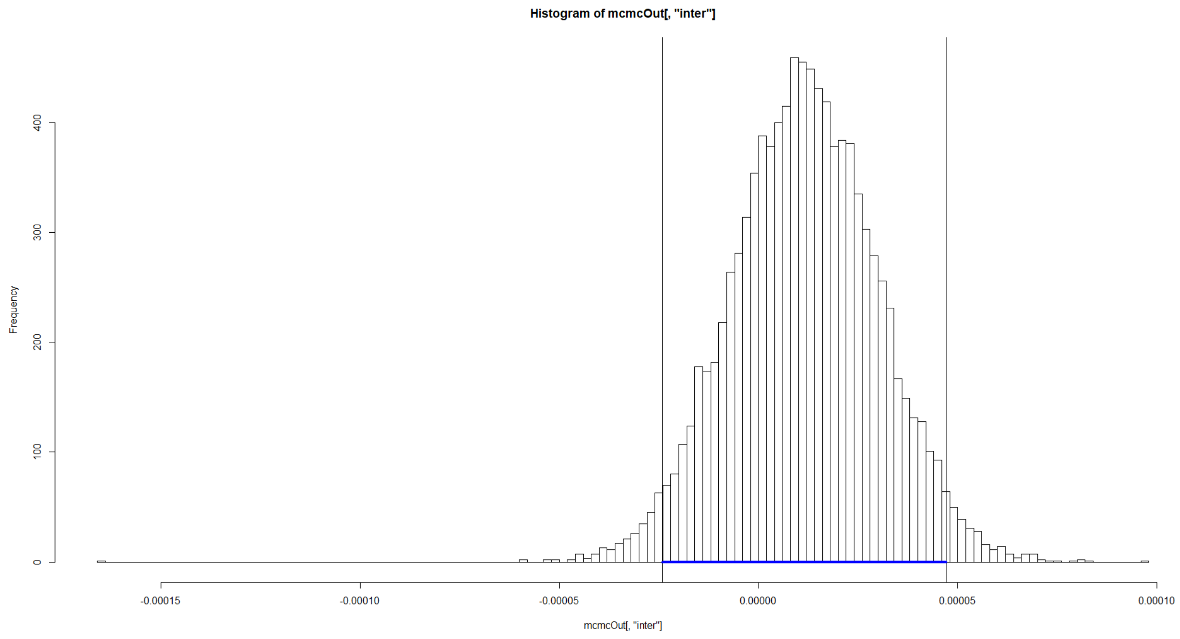

(B)

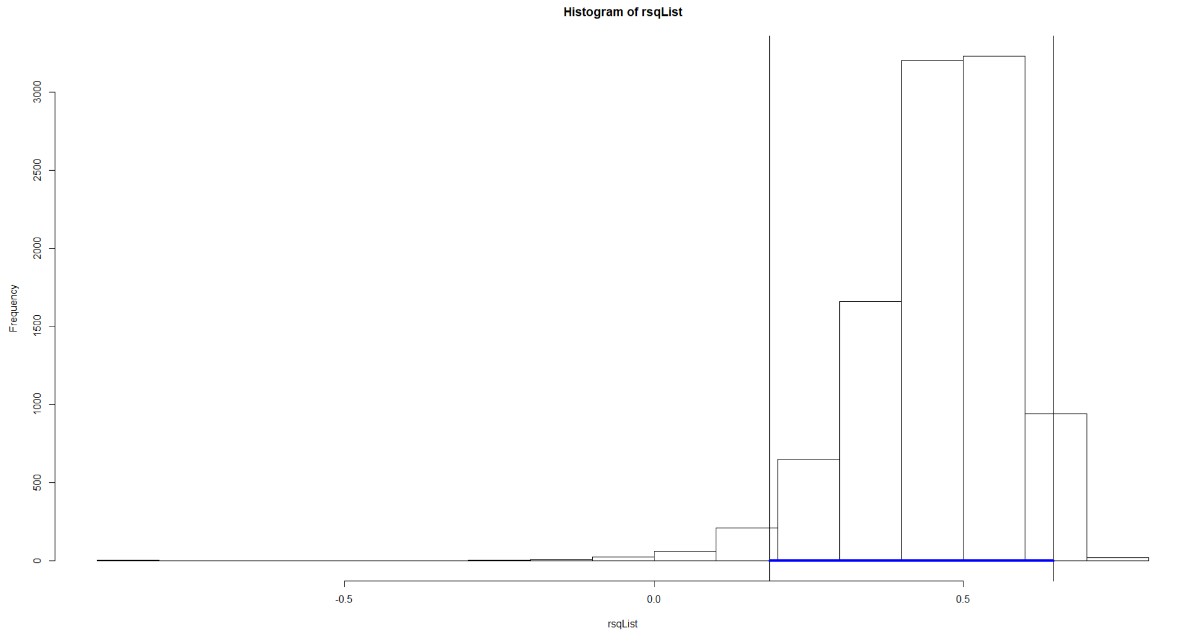

Supplementary Figure 6. Bayesian Analysis with 48 participants (removal of one potential outlier). (A) 95% HDI for the interaction regression coefficients. (B) 95% HDI for R^2.

(A)

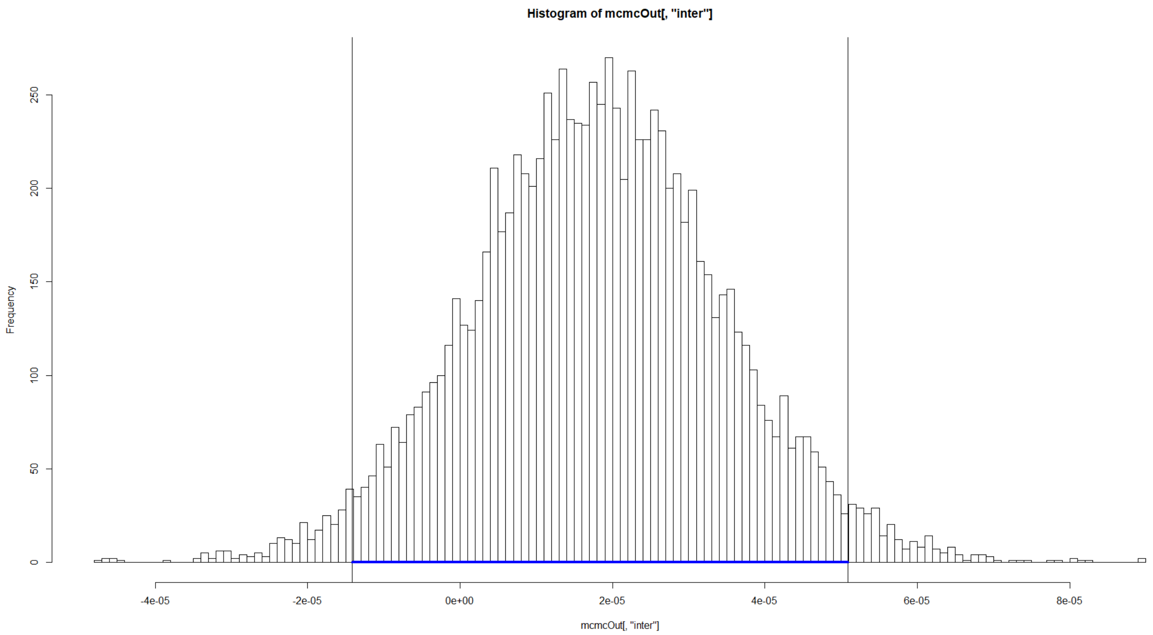

(B)

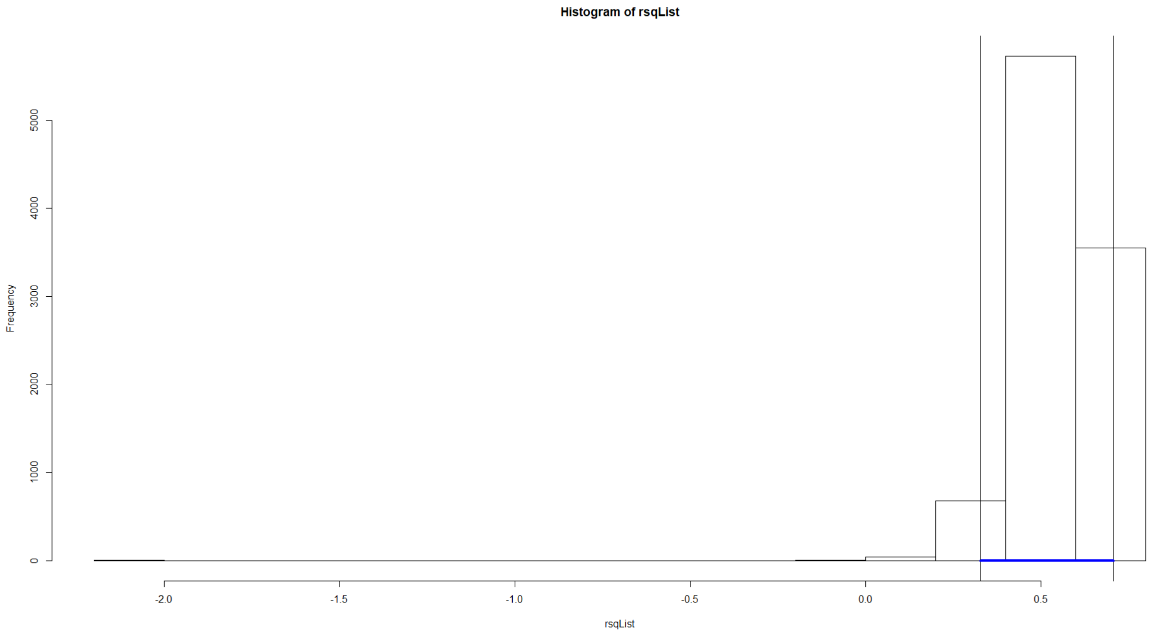

Supplementary Figure 7. Bayesian Analysis with 47 participants (removal of two potential outliers). (A) 95% HDI for the interaction regression coefficients. (B) 95% HDI for R^2.
